# Translational Control during Calicivirus Infection

**DOI:** 10.3390/v8040104

**Published:** 2016-04-20

**Authors:** Elizabeth Royall, Nicolas Locker

**Affiliations:** Faculty of Health and Medical Sciences, School of Biosciences and Medicine, University of Surrey, Guildford GU2 7HX, UK; e.royall@surrey.ac.uk

**Keywords:** calicivirus, VPg, reinitiation

## Abstract

In this review, we provide an overview of the strategies developed by caliciviruses to subvert or regulate the host protein synthesis machinery to their advantage. As intracellular obligate parasites, viruses strictly depend on the host cell resources to produce viral proteins. Thus, many viruses have developed strategies that regulate the function of the host protein synthesis machinery, often leading to preferential translation of viral mRNAs. Caliciviruses lack a 5′ cap structure but instead have a virus-encoded VPg protein covalently linked to the 5′ end of their mRNAs. Furthermore, they encode 2–4 open reading frames within their genomic and subgenomic RNAs. Therefore, they use alternative mechanisms for translation whereby VPg interacts with eukaryotic initiation factors (eIFs) to act as a proteinaceous cap-substitute, and some structural proteins are produced by reinitiation of translation events. This review discusses our understanding of these key mechanisms during caliciviruses infection as well as recent insights into the global regulation of eIF4E activity.

## 1. Caliciviruses: Genome and Translational Challenges

The *Caliciviridae* family comprises small RNA viruses of both medical and veterinary importance. Caliciviruses are classified into five genera: *Vesivirus*; *Lagovirus*; *Norovirus*; *Sapovirus*; and *Nebovirus* [1]. *Noroviruses* are, themselves, further grouped into six genogroups, GI–GVI of which GI, GII, and GIV infect humans. Human norovirus (HuNoV) is a leading cause of gastroenteritis worldwide, a severe economic burden to the health organisations of both the developed and developing world [2]. The World Health Organisation reported an estimated 120 million cases of norovirus worldwide including 35,000 deaths in 2010 [3]. Furthermore, sapoviruses and animal noroviruses also cause outbreaks of gastroenteritis in farmed animals, while feline calicivirus (FCV), a member of the *Vesivirus* genus, causes upper respiratory tract infections and lethal systemic disease in cats [4,5].

Despite recent studies indicating that limited HuNoV replication can occur in immortalised B cells, the mechanisms involved in translation and replication of the HuNoV genome have not yet been fully elucidated, owing to the lack of a robust cell culture system [6,7,8]. Our current knowledge is derived from other caliciviruses used as representative models, including feline calicivirus (FCV), murine norovirus (MNV), and porcine sapovirus (PSaV), due to both the development of reverse genetics systems and the availability of cell cultures for their propagation (reviewed in [9,10,11,12,13,14]).

The calicivirus genome consists of a non-segmented single-stranded, positive-sense RNA genome ranging from 6.7–8.5 kb [15,16,17]. While eukaryotic mRNAs commonly display a 7^Me^-GpppG cap structure, a viral genome-linked protein (VPg), ranging from 13–16 kDa, is covalently attached at the 5′ end the genomic RNA, which is polyadenylated at the 3′ end (*ibid*). In all members of the *Caliciviridae*, a 3′ co-terminal subgenomic RNA is also transcribed during infection. On both the calicivirus genomic and subgenomic RNAs, the 5′ VPg drives the initiation of translation.

**Figure 1:**
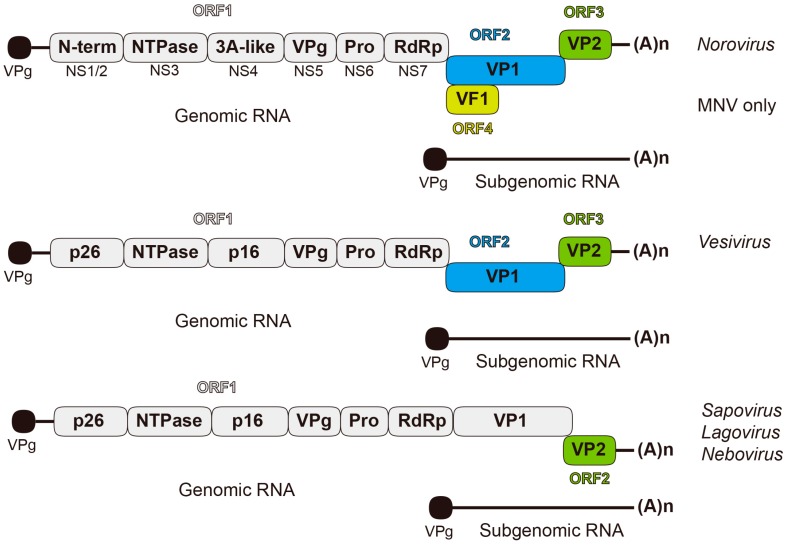
Diagrammatic representation of open-reading frame (ORF) usage in the different *Caliciviridae* genomic and subgenomic RNA. Protein VPg present at 5’-ends of both the genomic and subgenomic RNAs is depicted by a black circle at the end of a line representing the viral RNA. The full-length genome is organized into 2–4 ORFs. A subgenomic RNA consisting of ORF2 and ORF3 is also produced during replication. ORF1 encodes a polyprotein, processed into individual non-structural proteins, while ORF2 and ORF3 code for the structural capsid proteins VP1 and VP2, respectively. In addition to these core proteins, MNV ORF4 encodes VF1, and the FCV (Vesivirus) encodes a leader capsid (LC).

Following cell entry, the viral genome can readily be translated and acts as an mRNA template comprising of either two open reading frames (ORFs) for members of the *Lagovirus*, *Sapovirus*, and *Nebovirus* genera, or three ORFs for *Vesivirus* and *Norovirus* (Figure 1) [18]. Murine noroviruses also possess a fourth ORF encoding a virulence factor, VF1, involved in counteracting the host immune response [19]. ORF1 encodes a polyprotein processed co- and post-translationally by the virus-encoded protease into distinct non-structural proteins (Figure 1). The expression of the structural proteins, the major capsid protein VP1 (~60 kDa), and the minor capsid protein VP2 (8–22 kDa), differs among the genera. In *Vesivirus* and *Norovirus*, VP1 and VP2 are encoded by ORF2 and ORF3 from the subgenomic RNA; whereas in *Sapovirus*, *Lagovirus*, and *Nebovirus*, VP1 and VP2 are encoded by ORF1 and ORF2, respectively, and the VP1 coding region is located at the 3′end of ORF1. Within the bicistronic subgenomic calicivirus mRNAs the VP1 stop and the VP2 start codons typically overlap, but can be separated by a short stretch of 3–10 nucleotides. As such, the translation of the second cistron, VP2, occurs by a process of reinitiation allowing the translation of two proteins from one mRNA [20,21,22,23,24,25].

Eukaryotic mRNAs are mainly monocistronic. Protein synthesis usually begins with the assembly of the pre-initiation complex (PIC) at the 5′ methyl guanosine cap (reviewed in [26]). The 40S ribosomal subunit, and the eukaryotic initiation factor (eIF)2-guanosine triphosphate (GTP)/Met-tRNAi^Met^ ternary complex, stimulated by the initiation factors eIF1, eIF1A, and eIF5, come together with eIF3 to build the 43S PIC. Attachment of the 43S initiation complex at the 5′ cap takes place with the additional interaction of eIF4A, eIF4B, and eIF4F. The combined activities of eIFs 4A, 4B, and 4F promote the unwinding of the proximal RNA secondary structure enabling the 43S complex to scan the mRNA, until it locates the AUG start codon. Upon formation of the codon-anticodon base pairing within the peptidyl (P) site of the 40S subunit and formation of the 48S initiation complex, eIF5 and 5B facilitate 60S subunit joining to form elongation-competent 80S ribosomes. Caliciviruses use two major strategies, the presence of a 5′ terminal VPg protein, and the mechanism of reinitiation, to subvert the cellular protein synthesis machinery and maximize the coding potential of their genome.

## 2. VPg Protein Interactions and Their Role in Initiation

In several virus families, including Picornaviridae, Caliciviridae, and Potyviridae, the 5′ VPg protein acts as a primer for genome replication, however its role in translation is restricted to some of these viruses [27]. In caliciviruses, the VPg protein is covalently linked to the 5′ end of both the genomic and the subgenomic RNAs by a phosphodiester bond between the hydroxyl group of a tyrosine residue in VPg and a uridine or guanine at the 5′ end of the viral RNA [16,17,27,28,29,30]. The tyrosine residue is conserved throughout the caliciviruses within a conserved linkage site defined by the following D(E/D)EYDEΦ motif, where Φ is any aromatic residue [28,31,32,33,34]. The viral RNA-dependent RNA-polymerase can nucleotidylate the VPg protein, which is then extended to produce the RNA-linked VPg in either a template-dependent or -independent manner [28,32].

**Figure 2:**
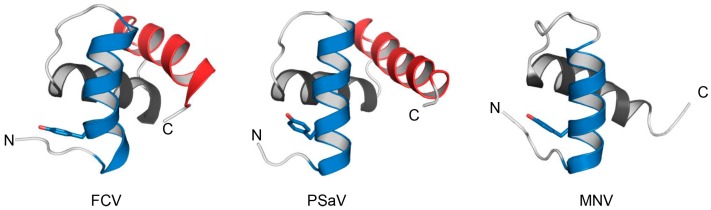
Solution structures of caliciviral VPgs. Cartoon representation of a representative model of each of the structured cores of the Feline Calicivirus (FCV), Porcine Sapovirus (PSV), and Murine Norovirus (MNV) VPg proteins. Each of these models have been deposited in the RCSB data base (accession numbers FCV: 24 MH, PSV: 2MXD, MNV: 2M4G) and are described in detail elsewhere [35,36]. The N-terminal, middle and C-terminal helices are coloured blue, black and red respectively. The side-chain of the proposed nucleotide accepting tyrosine is shown as sticks in each model.

The VPg proteins vary in size and sequence among caliciviruses, which indicates possibly large structural differences. The solution structures of VPg from representatives of three different genera, FCV (*Vesivirus*), PSaV (*Sapovirus*), and MNV (*Norovirus*), have all been determined by NMR spectroscopy and shown to adopt a compact helical core structure, flanked by flexible or disordered N and C-termini domains [35,36]. The VPg cores of FCV and PSaV comprise similar well-defined three-helix bundles (amino acids 12–72), whereas that of MNV is truncated containing only two helices (amino acids 23 to 55) that correspond to the first two helices of FCV and PSaV VPg as shown in Figure 2 [35,36]. The helical core contains the uridinylated and conserved tyrosine. Overall, the hydrophobic residues scaffolding the helical core structure of PSaV and FCV are largely conserved, and hydrophobic interactions between helices 2 and 3 stabilise the conformation. These core residues are also conserved in MNV, but the two-helix core is additionally stabilised by a salt bridge between residues R32 and D48 in helices 1 and 2, respectively. However despite these structural similarities, the VPg proteins from different caliciviruses behave differently in translation initiation, through a distinct network of interactions with eIFs.

VPg is required for the infectivity of the viral RNA transfected into cells and interacts with several host cellular factors involved in translation [16,37,38,39,40]. The removal of VPg from viral RNA impairs translation of the viral proteins and prevents viral replication [41,42]. In sapoviruses, vesiviruses, and noroviruses, the VPg proteins have been shown to interact with eIFs to mediate the initiation of translation and to date the best characterized interactions are with members of the eIF4F complex [43,44,45]. For members of the lagovirus and nebovirus genera, the translation mechanisms and VPg-mediated interactions have not yet been fully elucidated.

Using cap-sepharose pull-down and eIF4E-depletion with siRNA or 4EBP1 proteins, it was shown that the interaction of VPg with eIF4E is conserved among the *Caliciviridae* [40,43,45]. While this interaction is essential for initiation of translation in FCV [40], RHDV [46], and PSaV [45], MNV translation is not affected by the depletion of eIF4E nor by separation of the eIF4E-binding domain from eIF4G [43,44]. Thus, FCV and PSaV require an intact eIF4G-eIF4E interaction for efficient translation [43,45]. Indeed, the cleavage of eIF4G with Foot-and-mouth disease virus (FMDV) l-pro, to separate off the eIF4E binding site, inhibits translation in both viruses; as opposed to translation on MNV VPg-linked RNA where initiation can proceed normally. It is noteworthy that, while eIF4GI and eIF4GII are both cleaved following FCV infection, at 7 and 5 h post-infection, respectively [47], the cleavage products obtained do not separate the eIF4E binding site from the eIF3 binding site which is in keeping with the requirement of eIF4E for FCV translation. This eIF4GI/II cleavage is more likely related to host cell shut-off as it occurs late during the replication cycle, together with apoptosis and the release of virions [47,48]. This resemblance in behaviour between FCV and PSaV mRNAs tallies with the fact that PSaV and FCV VPg share a higher degree of sequence similarity. Furthermore, both FCV and PSaV translation are insensitive to the addition of cap analogues, suggesting the binding interface of eIF4E with VPg differs from that with a canonical cap [43,45].

The MNV VPg-eIF4G interaction was also identified using tandem-affinity purification of protein partners [44]. eIF4G is a large scaffolding initiation factor, with multiple domains mediating interactions with several other eIFs. Biochemical assays have demonstrated that VPg binds to the middle fragment of eIF4G (4GM; residues 652–1132) [44]. Recently, using pull-down assays, fluorescence anisotropy, and isothermal titration calorimetry (ITC), the exact interaction was mapped to the 20 C-terminal residues of the VPg (residues 104–124), which recruit the HEAT-1 domain within 4GM [49]. Ten residues in this eIF4G-binding motif region defined by MNV VPg are strictly conserved in all NV genogroups [49]. In contrast, FCV VPg has only 27% amino acid sequence identity with MNV VPg, and is therefore unable to bind either the HEAT-1 domain or 4GM. There is also significant conservation of the 4G-binding motif in the C-terminus of the human astrovirus type 4 VPg, but the astrovirus VPg does not bind to eIF4G HEAT-1 [49].

MNV, PSaV, and FCV RNA translation are all sensitive to inhibition by hippuristanol, a small-molecule inhibitor of eIF4A [43,50]. Furthermore, expression of a dominant-negative eIF4A mutant inhibited both MNV and FCV translation *in vitro* [43]. The binding site of MNV VPg to the HEAT-1 domain on eIF4G lies away from the eIF4A-binding region so the two binding sites are distinct. This could suggest that MNV VPg forms a ternary complex with eIF4G and eIF4A and correlates with the fact that it interacts with a functional eIF4F complex in the cell [44]. Given the very short 5′ UTR length in calicivirus genomes, mainly ranging from 4 to 20 nucleotides (with some longer up to 100), the functional requirement for eIF4A is questionable. However, previous studies have shown that the calicivirus 5′ end genomes are highly structured and, thus, eIF4A may participate in unfolding the viral RNA to facilitate start codon recognition [51]. Furthermore, while FCV VPg does not interact with PABP, which is cleaved by the FCV protease during infection, PABP interacts with MNV VPg, which could suggest a role in circularising the viral mRNA and promoting translation [40,44,52].

In addition to interactions with members of the eIF4F complex, Daughenbaugh *et al*. [38] were also able to demonstrate direct binding of eIF3 to the VPg from the prototype human norovirus Norwalk (NV). Furthermore, both the genomic and the subgenomic 5′ ends of FCV RNA can interact with the translation factor PTB [53]. During FCV infection, PTB relocates from the nucleus to the cytoplasm where it associates with the replication complex and suppresses translation later in the virus life-cycle, thereby regulating the mechanisms of translation and replication.

**Figure 3:**
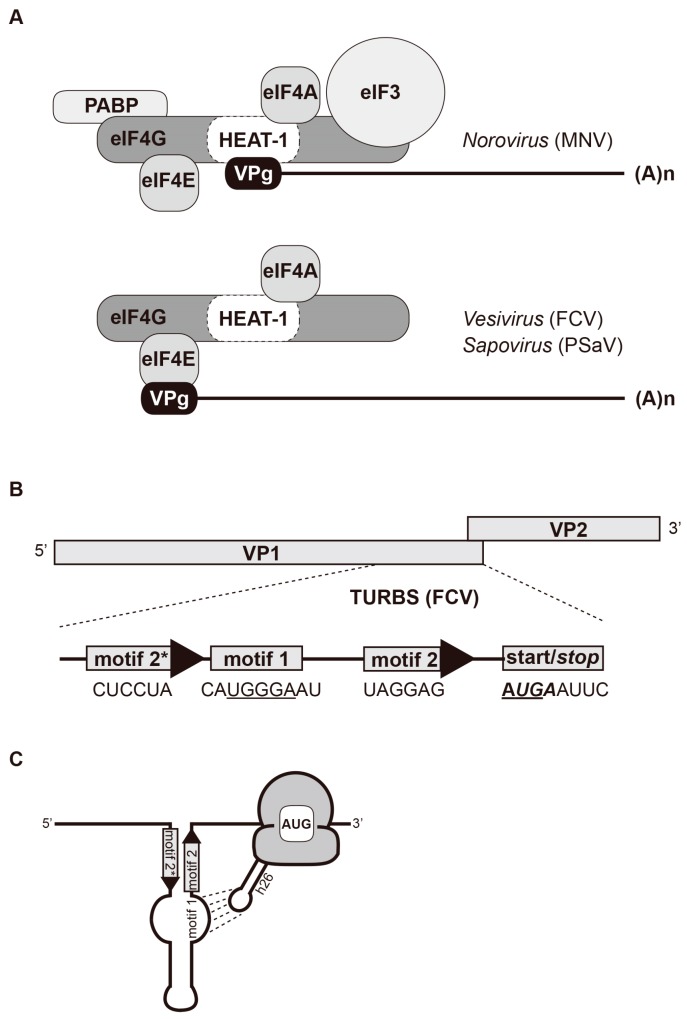
Interactions of caliciviral RNA and protein important in VPg-driven and reinitiation of translation. (**A**) Schematic representation of the interaction networks driving VPg-mediated translation. MNV VPg directly recruits eIF4G through binding the HEAT-1 domain; this is thought to facilitate the recruitment of additional translation factors including eIF4A, eIF4E, eIF3 and PAPB. FCV and PSaV VPg direct bind to eIF4E and interact with eIF4E and eIF4A; (**B**) Schematic representation of the FCV TURBS motif at the FV VP1/VP2 junction. The location of the individual motifs is indicated with the bases highlighted below. The underlines bases correspond to conserved motif 1 residue that have been proposed to base pair with the helix 26 of the 18S rRNA. The bold italic bases correspond to the VP1 stop codon while the bold underlined bases are the VP2 start codon; (**C**) Architecture of the FCV TURBS. The base pairing between motif 2 and 2* allows the formation of a long stem loop structure in which the motif 1 conserved residues are exposed in an internal loop. These bases pair with the helix 26 of the 18S rRNA (as depicted by dotted lines), which help docking the ribosome onto the viral mRNA, allowing reinitiation on the down-stream ORF.

Overall, the *caliciviridae* VPg proteins are able to behave as a proteinaceous 5′ cap substitute (Figure 3A). While the calicivirus and sapovirus VPg proteins require a direct binding with eIF4E, an interaction between eIF4E and eIF4G, and eIF4A activity, norovirus VPg recruits the eIF4G HEAT-1 domain and does not require an interaction with eIF4E [43,44,49]. Affinity for this key interaction is in the micromolar range, significantly weaker than that of the eukaryotic cap affinity for eIF4F that lies in the nanomolar range [54]; however, it is probably strengthened by the additional interactions reported between norovirus VPg and eIF4E or eIF3 [38]. The absence of eIF4G cleavage in cells infected with norovirus supports a model in which the entire eIF4F complex would be recruited to the VPg through essential direct interactions with either eIF4G or eIF4E. Further *in vitro* translation reconstitution studies will be required to dissect the requirement for eIFs during the assembly of elongation-competent ribosomes on calicivirus mRNAs.

## 3. Reinitiation Mechanisms in Calicivirus Translation

Upon recognition of stop codons, post-termination complexes can become split into free 60S and tRNA/mRNA-associated 40S subunits instead of being recycled. These 40S subunits may remain associated with the mRNA and reinitiate translation upstream or downstream at nearby AUGs, thereby resulting in a reinitiation event and translation of a second downstream reading frame [55,56]. Although the reinitiation event was first described in caliciviruses allowing the translation of VP2 from the bicistronic subgenomic mRNA encoding VP1 and VP2, it has also been found to occur in cellular mRNAs [57,58].

Overall, a reinitiation event is rare and depends on *cis*-acting sequences in RNA that tether the ribosome to the reinitiation site, whereas the basic mechanism of translation reinitiation is conserved throughout the caliciviruses, the efficiency of expression of the downstream cistron varies between 5% and 20% of the VP1 translation rate. Habeta *et al.* have shown that for FCV, the level of VP2 expression is directly affected by the frequency of translation reinitiation, which is regulated by the primary and secondary structure of the region downstream of the start/stop sites [59]. In caliciviruses, the process of termination/reinitiation is orchestrated by a 40–80 nt long element upstream of the restart VP2 AUG codon, designated as the termination upstream ribosome binding site (TURBS). In noroviruses, a second TURBS has been identified upstream of the VP1 coding region within the polyprotein gene of GIII bovine norovirus [60]. This second TURBS is also present in GII HuNoV but whether it drives VP1 translation remains unclear with contradictory reports [23,60]. Given that the expression of the VP1 from the subgenomic RNA is likely more effective, the contribution of this TURBS-mediated VP1 translation to the overall levels of VP1 is debatable. For HuNoV, unlike other caliciviruses, the VP1 stop codon overlaps with the VP2 start codon at the ORF2/ORF3 boundary: UA*AUG* (VP1 stop; *VP2 start*). For all other caliciviruses, the VP1-VP2 boundary, and the polymerase/VP1 border of HuNoV, are organised with the stop codon of the first ORF located downstream of the start codon of the second ORF, with a distance ranging from one to 14 nt.

Within the TURBS, the existence of essential motifs, namely motifs 1, 2, and 2*, was first reported for FCV and RHDV and later on for bovine, murine, and human norovirus (Figure 3B) [22,23,24]. Motif 1 contains a pentameric UGGGA core, conserved throughout the caliciviruses and located at similar positions within their mRNAs, upstream of the 3′ terminal ORF [59]. The UGGGA sequence is exposed in a loop and establishes intermolecular base paring with helix 26 (h26) of the 18S rRNA, thereby allowing the tethering of 40S subunits to the viral RNA (Figure 3C). Supporting this model, mutations within motif 1 that reduce reinitiation efficacy can be compensated by compensatory changes in the 18S rRNA sequence [22]. Motif 2 and 2* correspond to the species-specific primary sequence elements, complementary to one another. Located 12 to 23 nt upstream of the reinitiation AUG, the annealing of motif 2 and 2* establishes a secondary structure that positions the motif 1-bound ribosome relative to the restart site of the downstream ORF (Figure 3C) [20,21].

While eIF3 has been shown to play a major role in translation termination and ribosome recycling, the TURBS strongly increases the efficiency of reinitiation by recycled 40S subunits reducing the dependence on eIF3 [56,61]. This might be critical for reinitiation after translation of long open reading frames, such as FCV ORF1, when the transient association of eIF3 with the elongating ribosomes is probably no longer occurring. The ribosome might be held at the stop/restart region by base pairing of TURBS with h26 of the 18S rRNA until it could be stabilised by subsequent binding of eIF3, which might also enhance recruitment of eIF2-termination complexes [25,61]. Furthermore, using *in vitro* reconstitution and toe-printing to examine the formation of ribosomal complexes at the RhDV ORF2 restart codon of VP2 and NV ORF2 restart codon of VP1, post-termination ribosomes were found to rebind initiator tRNA efficiently and move 2 nt upstream or 5 nt downstream, respectively, to reinitiate at the ORF2 AUG [61]. The TURBS-dependent reinitiation event by 40S subunits does not require eIF3, but eIF3 can stimulate reinitiation if eIF1 or eIF1A are present individually [61]. Moreover, eIF2, 1, and 1A were found to be sufficient for reinitiation, emphasising the involvement of 40S subunits [61]. Mutations within the TURBS element that disrupt the secondary structure were also found to increase the dependence on eIF3 for reinitiation [25]. Thus, while eIF3 is not strictly required for 40S recruitment, further structural studies may elucidate how the TURBS/eIF3 and TURBS/40S interactions contribute to the reinitiation mechanism by manipulating the ribosome conformation and the assembly of translating ribosomes.

Most studies seem to indicate that the site at which reinitiation occurs is neither linked to the termination site, nor relies on the presence of an AUG codon. Furthermore, the reinitiation frequency generally decreases as the distance between the start and stop sites increases. The distance from the secondary structure formed by motifs 2/2* would appear to define the start site by positioning the motif 1-bound post-termination ribosome within a specific distance from the TURBS structure. This distance ranges from 12 to 24 nt in different caliciviruses and will depend more specifically on the exact position of the P-site of the ribosome on the mRNA. A potential advantage is that it might obviate the need for a good initiation codon context as found with scanning-dependent initiation [62]. Indeed, Luttermann *et al.* [23] found that translation at the VP1 termination signal close to the VP2 start site is important for efficient VP2 translation. However, the AUG re-start codon was not essential as reinitiation could occur on non-AUG codons, albeit at lower levels.

## 4. Regulation of eIF4E Activity during Calicivirus Infection

As discussed previously, caliciviruses have evolved several strategies to hijack cellular eIFs and mediate viral protein synthesis through VPg-mediated initiation and TURBS-mediated reinitiation of translation. All these mechanisms require intricate interactions between viral proteins, or RNA, with ribosomal subunits and eIFs. In addition, it is well documented that viruses can modulate translational activity by altering the function of eIFs. For example, the phosphorylation of eIF2α following the recognition of RNA replication intermediates, or the cleavage of eIF4G by viral proteases, both result in translational shut-off [63,64]. Among the translation factors, the regulation of the cap-binding protein eIF4E is critical and limiting for translation [65]. The hypophosphorylated eIF4E-binding proteins (4E-BPs) can sequester eIF4E by displacing eIF4G [66]. Upon stimulation of the phosphatidylinositol 3′ kinase-Akt-mammalian target of rapamycin (PI3K-Akt-mTOR) pathway the phosphorylation of 4E-BP impedes the interaction with eIF4E, thereby stimulating eIF4F complex assembly and translation [67,68]. Viruses, for example poliovirus and vesicular stomatitis virus, can induce the dephosphorylation of 4E-BPs to reduce eIF4E availability and impair cellular translation [64,69,70].

The phosphorylation of eIF4E at S209 can also impact on its activity. The signalling cascade through the mitogen-associated protein kinase (MAPK) pathway, culminates in the activation of p38 and ERK1/2, which both can phosphorylate the eIF4E kinase Mnk1/2 [71,72]. Despite conflicting evidence regarding the impact of eIF4E phosphorylation on its affinity for the cap, it is becoming increasingly evident that p-eIF4E controls the translation of specific mRNAs encoding proteins associated with cell proliferation, inflammation, and interferon production [73,74,75]. Given that infections with herpes simplex virus 1 and human cytomegalovirus both lead to an accumulation of phosphorylated eIF4E; while influenza virus, poliovirus, and encephalomyocarditis virus induce eIF4E dephosphorylation (reviewed in [63]), it seemed reasonable to postulate that viruses can reprogramme gene expression in the infected host via p-eIF4E. Recently, we demonstrated that MNV infection modulates the MAPK pathway to trigger eIF4E phosphorylation [76]. Our results also showed that p38-driven Mnk1/2 activation during MNV infection is required for virus replication. Furthermore, p-eIF4E associates with translating ribosomes during infection, resulting in translational upregulation of a subset of cellular mRNAs, such as the NFκB inhibitor *Nfkbia* and ribosomal proteins. Therefore, we hypothesized that during MNV infection, the phosphorylation of eIF4E induces global changes in the translational landscape of the infected cells and this plays a role in the control of the antiviral response. The exact extent of this mechanism is the subject of ongoing investigation within our group. In addition, the degree of conservation of this phenomenon within the *Caliciviridae* family is unknown. Furthermore, given that eIF4E is part of the VPg-driven viral translation initiation complex, it remains unclear how eIF4E phosphorylation impacts on the recruitment of initiation factors by VPg and on the ability of Mnk1/2 to access eIF4E when both eIF4E and Mnk1/2 are bound to eIF4G [77,78,79].

## 5. Conclusions and Future Directions

The caliciviruses have evolved to maximise the genetic potential of their limited genome to express a comprehensive range of functional and adaptive proteins. Most studies to date have aimed at dissecting the network of interactions mediated by the caliciviruses VPg and the identification of the determinants required to mediate reinitiation events. These have resulted in invaluable mechanistic insights in the translation of viral proteins from calicivirus genomic and subgenomic mRNAs. Several questions however remain to be addressed including (i) what are the eIFs required to mediate the assembly of elongation-competent ribosomes on calicivirus mRNA; (ii) given current advances in our understanding of translation through structural studies, can we elucidate the structure of the VPg-driven initiation complexes and TURBS-40S complexes, thus, increasing our understanding of calicivirus translation. Moreover, our insights on the regulation of eIF4E phosphorylation raise questions related to (i) the role of p-eIF4E on VPg-mediated viral translation; (ii) the global extend of cellular mRNA translational reprogramming and (iii) the role of the mTOR pathway in controlling eIF4E availability during calicivirus infection. Finally, given the differences observed thus far between members of the *Caliciviridae* family, a conserved role for p-eIF4E during infection by different caliciviruses remain to be established.

It is known that infection by viruses imposes major stress on the host cell. In response to this stress, infected cells can induce several defense mechanisms, these include a global reduction in host protein synthesis to promote cell survival and limit the use of energy and nutrients [80]. This process is triggered by the activation of stress-activated kinases, mainly the protein kinase R (PKR), but also the PKR-like ER-localized kinase (PERK), which results in the phosphorylation of the eukaryotic initiation factor 2α (eIF2 α) [80]. This in turn prevents the recycling of the ternary complex tRNAiMet-GTP-eIF2 and the delivery of the initiator tRNA to the ribosome, thereby stalling the initiation of translation and resulting in a shutdown of protein synthesis [81,82,83]. Following this translational arrest, mRNAs contained within stalled ribosome complexes accumulate in cytoplasmic structures called stress granules (SGs) [81,84]. The resulting stoppage in protein synthesis following SG assembly is problematic for viruses as they rely on the host cell protein synthesis machinery for production of their proteins. Consequently several viruses have evolved different strategies to ensure that viral mRNA translation can continue by disrupting SG formation during infection or by exploiting SGs for their replication [85,86,87]. Currently little to nothing is known about the control of eIF2α phosphorylation during calicivirus infection and the consequences on SGs assembly. Current studies by our and other teams should lead to a better understanding of the relationship between caliciviruses and SGs.

Overall, despite significant advances within the past decade in our understanding of calicivirus regulation of translation, several unanswered questions remain to be addressed to fully dissect calicivirus-host interactions that regulate protein synthesis in the infected host. In addition, the development of new models to propagate human norovirus will undoubtedly spark further studies to revisit and complement our current knowledge of calicivirus biology.

## References

[1] Carstens E.B. (2010). Ratification vote on taxonomic proposals to the international committee on taxonomy of viruses (2009). Arch. Virol..

[2] Pringle K., Lopman B., Vega E., Vinje J., Parashar U.D., Hall A.J. (2015). Noroviruses: Epidemiology, immunity and prospects for prevention. Future Microbiol..

[3] Havelaar A.H., Kirk M.D., Torgerson P.R., Gibb H.J., Hald T., Lake R.J., Praet N., Bellinger D.C., de Silva N.R., Gargouri N. (2015). World health organization global estimates and regional comparisons of the burden of foodborne disease in 2010. PLoS Med..

[4] Desselberger U., Goodfellow I. (2014). Noroviruses: A global cause of acute gastroenteritis. Lancet Infect. Dis..

[5] Radford A.D., Addie D., Belak S., Boucraut-Baralon C., Egberink H., Frymus T., Gruffydd-Jones T., Hartmann K., Hosie M.J., Lloret A. (2009). Feline calicivirus infection. Abcd guidelines on prevention and management. J. Feline Med. Surg..

[6] Duizer E., Schwab K.J., Neill F.H., Atmar R.L., Koopmans M.P., Estes M.K. (2004). Laboratory efforts to cultivate noroviruses. J. Gen. Virol..

[7] Jones M.K., Grau K.R., Costantini V., Kolawole A.O., de Graaf M., Freiden P., Graves C.L., Koopmans M., Wallet S.M., Tibbetts S.A. (2015). Human norovirus culture in B cells. Nat. Protoc..

[8] Papafragkou E., Hewitt J., Park G.W., Greening G., Vinje J. (2014). Challenges of culturing human norovirus in three-dimensional organoid intestinal cell culture models. PLoS ONE.

[9] Thorne L.G., Goodfellow I.G. (2014). Norovirus gene expression and replication. J. Gen. Virol..

[10] Vashist S., Bailey D., Putics A., Goodfellow I. (2009). Model systems for the study of human norovirus biology. Future Virol..

[11] Katayama K., Murakami K., Sharp T.M., Guix S., Oka T., Takai-Todaka R., Nakanishi A., Crawford S.E., Atmar R.L., Estes M.K. (2014). Plasmid-based human norovirus reverse genetics system produces reporter-tagged progeny virus containing infectious genomic RNA. Proc. Natl. Acad. Sci. USA.

[12] Chang K.O., Sosnovtsev S.V., Belliot G., Wang Q., Saif L.J., Green K.Y. (2005). Reverse genetics system for porcine enteric calicivirus, a prototype *Sapovirus* in the *Caliciviridae*. J. Virol..

[13] Liu G., Ni Z., Yun T., Yu B., Chen L., Zhao W., Hua J., Chen J. (2008). A DNA-launched reverse genetics system for rabbit hemorrhagic disease virus reveals that the VP2 protein is not essential for virus infectivity. J. Gen. Virol..

[14] Oka T., Takagi H., Tohya Y. (2014). Development of a novel single step reverse genetics system for feline calicivirus. J. Virol. Methods.

[15] Black D.N., Burroughs J.N., Harris T.J., Brown F. (1978). The structure and replication of calicivirus RNA. Nature.

[16] Herbert T.P., Brierley I., Brown T.D. (1997). Identification of a protein linked to the genomic and subgenomic mRNAs of feline calicivirus and its role in translation. J. Gen. Virol..

[17] Schaffer F.L., Ehresmann D.W., Fretz M.K., Soergel M.I. (1980). A protein, VPg, covalently linked to 36S calicivirus RNA. J. Gen. Virol..

[18] Rohayem J., Bergmann M., Gebhardt J., Gould E., Tucker P., Mattevi A., Unge T., Hilgenfeld R., Neyts J. (2010). Antiviral strategies to control calicivirus infections. Antivir. Res..

[19] McFadden N., Bailey D., Carrara G., Benson A., Chaudhry Y., Shortland A., Heeney J., Yarovinsky F., Simmonds P., Macdonald A. (2011). Norovirus regulation of the innate immune response and apoptosis occurs via the product of the alternative open reading frame 4. PLoS Pathog..

[20] Meyers G. (2007). Characterization of the sequence element directing translation reinitiation in RNA of the calicivirus rabbit hemorrhagic disease virus. J. Virol..

[21] Luttermann C., Meyers G. (2007). A bipartite sequence motif induces translation reinitiation in feline calicivirus RNA. J. Biol. Chem..

[22] Luttermann C., Meyers G. (2009). The importance of inter- and intramolecular base pairing for translation reinitiation on a eukaryotic bicistronic mRNA. Genes Dev..

[23] Luttermann C., Meyers G. (2014). Two alternative ways of start site selection in human norovirus reinitiation of translation. J. Biol. Chem..

[24] Napthine S., Lever R.A., Powell M.L., Jackson R.J., Brown T.D., Brierley I. (2009). Expression of the VP2 protein of murine norovirus by a translation termination-reinitiation strategy. PLoS ONE.

[25] Poyry T.A., Kaminski A., Connell E.J., Fraser C.S., Jackson R.J. (2007). The mechanism of an exceptional case of reinitiation after translation of a long ORF reveals why such events do not generally occur in mammalian mRNA translation. Genes Dev..

[26] Hinnebusch A.G. (2011). Molecular mechanism of scanning and start codon selection in eukaryotes. Microbiol. Mol. Biol. Rev..

[27] Goodfellow I. (2011). The genome-linked protein VPg of vertebrate viruses—A multifaceted protein. Curr. Opin. Virol..

[28] Machin A., Martin Alonso J.M., Parra F. (2001). Identification of the amino acid residue involved in rabbit hemorrhagic disease virus VPg uridylylation. J. Biol. Chem..

[29] Meyers G., Wirblich C., Thiel H.J. (1991). Genomic and subgenomic RNAs of rabbit hemorrhagic disease virus are both protein-linked and packaged into particles. Virology.

[30] Sosnovtsev S.V., Green K.Y. (2000). Identification and genomic mapping of the ORF3 and VPg proteins in feline calicivirus virions. Virology.

[31] Belliot G., Sosnovtsev S.V., Chang K.O., McPhie P., Green K.Y. (2008). Nucleotidylylation of the VPg protein of a human norovirus by its proteinase-polymerase precursor protein. Virology.

[32] Han K.R., Choi Y., Min B.S., Jeong H., Cheon D., Kim J., Jee Y., Shin S., Yang J.M. (2010). Murine norovirus-1 3Dpol exhibits RNA-dependent RNA polymerase activity and nucleotidylylates on Tyr of the VPg. J. Gen. Virol..

[33] Mitra T., Sosnovtsev S.V., Green K.Y. (2004). Mutagenesis of tyrosine 24 in the VPg protein is lethal for feline calicivirus. J. Virol..

[34] Subba-Reddy C.V., Goodfellow I., Kao C.C. (2011). VPg-primed RNA synthesis of norovirus RNA-dependent RNA polymerases by using a novel cell-based assay. J. Virol..

[35] Hwang H.J., Min H.J., Yun H., Pelton J.G., Wemmer D.E., Cho K.O., Kim J.S., Lee C.W. (2015). Solution structure of the porcine *Sapovirus* VPg core reveals a stable three-helical bundle with a conserved surface patch. Biochem. Biophys. Res. Commun..

[36] Leen E.N., Kwok K.Y., Birtley J.R., Simpson P.J., Subba-Reddy C.V., Chaudhry Y., Sosnovtsev S.V., Green K.Y., Prater S.N., Tong M. (2013). Structures of the compact helical core domains of feline calicivirus and murine norovirus VPg proteins. J. Virol..

[37] Burroughs J.N., Brown F. (1978). Presence of a covalently linked protein on calicivirus RNA. J. Gen. Virol..

[38] Daughenbaugh K.F., Fraser C.S., Hershey J.W., Hardy M.E. (2003). The genome-linked protein VPg of the norwalk virus binds eIF3, suggesting its role in translation initiation complex recruitment. EMBO J..

[39] Dunham D.M., Jiang X., Berke T., Smith A.W., Matson D.O. (1998). Genomic mapping of a calicivirus VPg. Arch. Virol..

[40] Goodfellow I., Chaudhry Y., Gioldasi I., Gerondopoulos A., Natoni A., Labrie L., Laliberte J.F., Roberts L. (2005). Calicivirus translation initiation requires an interaction between VPg and eIF4E. EMBO Rep..

[41] Sosnovtsev S., Green K.Y. (1995). RNA transcripts derived from a cloned full-length copy of the feline calicivirus genome do not require VPg for infectivity. Virology.

[42] Guix S., Asanaka M., Katayama K., Crawford S.E., Neill F.H., Atmar R.L., Estes M.K. (2007). Norwalk virus RNA is infectious in mammalian cells. J. Virol..

[43] Chaudhry Y., Nayak A., Bordeleau M.E., Tanaka J., Pelletier J., Belsham G.J., Roberts L.O., Goodfellow I.G. (2006). Caliciviruses differ in their functional requirements for eIF4F components. J. Biol. Chem..

[44] Chung L., Bailey D., Leen E.N., Emmott E.P., Chaudhry Y., Roberts L.O., Curry S., Locker N., Goodfellow I.G. (2014). Norovirus translation requires an interaction between the C terminus of the genome-linked viral protein VPg and eukaryotic translation initiation factor 4G. J. Biol. Chem..

[45] Hosmillo M., Chaudhry Y., Kim D.S., Goodfellow I., Cho K.O. (2014). *Sapovirus* translation requires an interaction between VPg and the cap binding protein eIF4E. J. Virol..

[46] Zhu J., Wang B., Miao Q., Tan Y., Li C., Chen Z., Guo H., Liu G. (2015). Viral genome-linked protein (VPg) is essential for translation initiation of rabbit hemorrhagic disease virus (RHDV). PLoS ONE.

[47] Willcocks M.M., Carter M.J., Roberts L.O. (2004). Cleavage of eukaryotic initiation factor eIF4G and inhibition of host-cell protein synthesis during feline calicivirus infection. J. Gen. Virol..

[48] Al-Molawi N., Beardmore V.A., Carter M.J., Kass G.E., Roberts L.O. (2003). Caspase-mediated cleavage of the feline calicivirus capsid protein. J. Gen. Virol..

[49] Leen E.N., Sorgeloos F., Correia S., Chaudhry Y., Cannac F., Pastore C., Xu Y., Graham S.C., Matthews S.J., Goodfellow I.G. (2016). A conserved interaction between a C-terminal motif in norovirus VPg and the heat-1 domain of eIF4G is essential for translation initiation. PLoS Pathog..

[50] Hosmillo M., Sweeney T.R., Chaudhry Y., Leen E., Curry S., Goodfellow I., Cho K.O. (2016). The RNA Helicase eIF4A is required for *Sapovirus* translation. J. Virol..

[51] Alhatlani B., Vashist S., Goodfellow I. (2015). Functions of the 5′ and 3′ ends of calicivirus genomes. Virus Res..

[52] Kuyumcu-Martinez M., Belliot G., Sosnovtsev S.V., Chang K.O., Green K.Y., Lloyd R.E. (2004). Calicivirus 3C-like proteinase inhibits cellular translation by cleavage of poly(a)-binding protein. J. Virol..

[53] Karakasiliotis I., Vashist S., Bailey D., Abente E.J., Green K.Y., Roberts L.O., Sosnovtsev S.V., Goodfellow I.G. (2010). Polypyrimidine tract binding protein functions as a negative regulator of feline calicivirus translation. PLoS ONE.

[54] Kaye N.M., Emmett K.J., Merrick W.C., Jankowsky E. (2009). Intrinsic RNA binding by the eukaryotic initiation factor 4F depends on a minimal RNA length but not on the m^7^G cap. J. Biol. Chem..

[55] Jackson R.J., Hellen C.U., Pestova T.V. (2012). Termination and post-termination events in eukaryotic translation. Adv. Protein Chem. Struct. Biol..

[56] Pisarev A.V., Hellen C.U., Pestova T.V. (2007). Recycling of eukaryotic posttermination ribosomal complexes. Cell.

[57] Gould P.S., Dyer N.P., Croft W., Ott S., Easton A.J. (2014). Cellular mRNAs access second ORFs using a novel amino acid sequence-dependent coupled translation termination-reinitiation mechanism. RNA.

[58] Powell M.L. (2010). Translational termination-reinitiation in RNA viruses. Biochem. Soc. Trans..

[59] Habeta M., Luttermann C., Meyers G. (2014). Feline calicivirus can tolerate gross changes of its minor capsid protein expression levels induced by changing translation reinitiation frequency or use of a separate VP2-coding mRNA. PLoS ONE.

[60] McCormick C.J., Salim O., Lambden P.R., Clarke I.N. (2008). Translation termination reinitiation between open reading frame 1 (ORF1) and ORF2 enables capsid expression in a bovine norovirus without the need for production of viral subgenomic RNA. J. Virol..

[61] Zinoviev A., Hellen C.U., Pestova T.V. (2015). Multiple mechanisms of reinitiation on bicistronic calicivirus mRNAs. Mol. Cell.

[62] Kozak M. (1989). Context effects and inefficient initiation at non-AUG codons in eucaryotic cell-free translation systems. Mol. Cell. Biol..

[63] Walsh D., Mathews M.B., Mohr I. (2013). Tinkering with translation: Protein synthesis in virus-infected cells. Cold Spring Harb. Perspect. Biol..

[64] Walsh D., Mohr I. (2011). Viral subversion of the host protein synthesis machinery. Nat. Rev. Microbiol..

[65] Duncan R., Milburn S.C., Hershey J.W. (1987). Regulated phosphorylation and low abundance of HeLa cell initiation factor eIF-4F suggest a role in translational control. Heat shock effects on eIF-4F. J. Biol. Chem..

[66] Mader S., Lee H., Pause A., Sonenberg N. (1995). The translation initiation factor eIF-4E binds to a common motif shared by the translation factor eIF-4 gamma and the translational repressors 4E-binding proteins. Mol. Cell. Biol..

[67] Clemens M.J. (2001). Translational regulation in cell stress and apoptosis. Roles of the eIF4E binding proteins. J. Cell. Mol. Med..

[68] Gingras A.C., Raught B., Gygi S.P., Niedzwiecka A., Miron M., Burley S.K., Polakiewicz R.D., Wyslouch-Cieszynska A., Aebersold R., Sonenberg N. (2001). Hierarchical phosphorylation of the translation inhibitor 4E-BP1. Genes Dev..

[69] Buchkovich N.J., Yu Y., Zampieri C.A., Alwine J.C. (2008). The TORrid affairs of viruses: Effects of mammalian DNA viruses on the PI3K-Akt-mTOR signalling pathway. Nat. Rev. Microbiol..

[70] Clemens M.J. (2005). Translational control in virus-infected cells: Models for cellular stress responses. Semin. Cell Dev. Biol..

[71] Morley S.J. (1997). Signalling through either the p38 or ERK mitogen-activated protein (MAP) kinase pathway is obligatory for phorbol ester and T cell receptor complex (TCR-CD3)-stimulated phosphorylation of initiation factor (eIF) 4E in Jurkat T cells. FEBS Lett..

[72] Waskiewicz A.J., Flynn A., Proud C.G., Cooper J.A. (1997). Mitogen-activated protein kinases activate the serine/threonine kinases Mnk1 and Mnk2. EMBO J..

[73] Furic L., Rong L., Larsson O., Koumakpayi I.H., Yoshida K., Brueschke A., Petroulakis E., Robichaud N., Pollak M., Gaboury L.A. (2010). eIF4E phosphorylation promotes tumorigenesis and is associated with prostate cancer progression. Proc. Natl. Acad. Sci. USA.

[74] Herdy B., Jaramillo M., Svitkin Y.V., Rosenfeld A.B., Kobayashi M., Walsh D., Alain T., Sean P., Robichaud N., Topisirovic I. (2012). Translational control of the activation of transcription factor NF-κb and production of type I interferon by phosphorylation of the translation factor eIF4E. Nat. Immunol..

[75] Ueda T., Watanabe-Fukunaga R., Fukuyama H., Nagata S., Fukunaga R. (2004). Mnk2 and Mnk1 are essential for constitutive and inducible phosphorylation of eukaryotic initiation factor 4E but not for cell growth or development. Mol. Cell. Biol..

[76] Royall E., Doyle N., Abdul-Wahab A., Emmott E., Morley S.J., Goodfellow I., Roberts L.O., Locker N. (2015). Murine norovirus 1 (MNV1) replication induces translational control of the host by regulating eIF4E activity during infection. J. Biol. Chem..

[77] Müller D.L.C., El Khawand S, Alard A., Schneider R.J., Bousquet C., Pyronnet S., Martineau Y. (2013). 4E-BP restrains eIF4E phosphorylation. Translation.

[78] Pyronnet S. (2000). Phosphorylation of the cap-binding protein eIF4E by the MAPK-activated protein kinase Mnk1. Biochem. Pharmacol..

[79] Pyronnet S., Imataka H., Gingras A.C., Fukunaga R., Hunter T., Sonenberg N. (1999). Human eukaryotic translation initiation factor 4G (eIF4G) recruits Mnk1 to phosphorylate eIF4E. EMBO J..

[80] Holcik M., Sonenberg N. (2005). Translational control in stress and apoptosis. Nat. Rev. Mol. Cell Biol..

[81] Anderson P., Kedersha N. (2008). Stress granules: The Tao of RNA triage. Trends Biochem. Sci..

[82] Garcia M.A., Meurs E.F., Esteban M. (2007). The dsRNA protein kinase PKR: Virus and cell control. Biochimie.

[83] Wek R.C., Jiang H.Y., Anthony T.G. (2006). Coping with stress: eIF2 kinases and translational control. Biochem. Soc. Trans..

[84] Anderson P., Kedersha N. (2002). Stressful initiations. J. Cell Sci..

[85] Montero H., Trujillo-Alonso V. (2011). Stress granules in the viral replication cycle. Viruses.

[86] Reineke L.C., Lloyd R.E. (2013). Diversion of stress granules and P-bodies during viral infection. Virology.

[87] Valiente-Echeverria F., Melnychuk L., Mouland A.J. (2012). Viral modulation of stress granules. Virus Res..

